# Role of the choroidal vascularity index in branch retinal vein occlusion (BRVO) with macular edema

**DOI:** 10.1371/journal.pone.0258728

**Published:** 2021-10-21

**Authors:** Bo-Een Hwang, Mirinae Kim, Young-Hoon Park

**Affiliations:** 1 Department of Ophthalmology and Visual Science, Seoul St. Mary’s Hospital, College of Medicine, The Catholic University of Korea, Seoul, Korea; 2 Catholic Institute for Visual Science, College of Medicine, The Catholic University of Korea, Seoul, Korea; Nicolaus Copernicus University, POLAND

## Abstract

**Purpose:**

To assess choroidal vasculature changes in eyes with branch retinal vein occlusion (BRVO) and macular edema (ME) using the choroidal vascularity index (CVI) and evaluate the effectiveness of CVI as a prognostic biomarker.

**Methods:**

35 patients with monocular BRVO and ME were analyzed retrospectively. Luminal and stromal areas in choroids of swept-source optical coherence tomography were calculated using the image binarization technique. The CVI was calculated as the ratio of the luminal to total choroidal area. The CVI of BRVO and ME eyes were compared with that of the unaffected fellow and post anti-vascular endothelial growth factor (VEGF) injected eyes. A regression analysis was performed on the choroidal parameters, logMAR visual acuity (VA) two years post disease onset and central macula thickness (CMT).

**Results:**

The CVI of BRVO and ME eyes was significantly lower than the fellow and post-injected eyes (p<0.05). The regression analysis showed a strong association between two years after logMAR VA and the CVI of fellow eyes (R^2^ = 0.433, p<0.001). Remarkable correlations were observed in the CVI and subfoveal choroidal thickness of BRVO and ME eyes (R^2^ = 0.189, 0.155, respectively, p<0.05). The CMT of diseased eyes were also significantly associated with the CVI of unaffected fellow eyes (R^2^ = 0.113, p<0.05).

**Conclusions:**

The alteration of CVI in BRVO and ME suggests that choroidal vasculature might be affected by extracellular fluid shift and VEGF changes. The fellow eye CVI could be a useful supplementary prognostic biomarker.

## Introduction

Branch retinal vein occlusion (BRVO) is a mechanical vascular obstructive disease characterized by retinal hemorrhage, macular edema (ME), or neovascularization [1]. Decreased visual acuity (VA) mainly occurs due to ME depending on the degree of the ischemic condition. The pathogenesis of ME in BRVO has been shown to be due to the increased concentration of vascular endothelial growth factor (VEGF) in retinal ischemia altering the inner blood-retina barrier (BRB) structure, causing fluid shift from the vessel components to the retinal cellular components [2]. As sustained macular edema can result in permanent visual disturbance, clinicians should ensure timely treatment with intraocular anti-VEGFs or steroids [3, 4].

Besides, the advent of swept-source optical coherence tomography (SS-OCT) and enhanced depth imaging spectral-domain OCT (EDI-OCT) has enabled ophthalmologists to perform quantitative measurements of the choroidal area, resulting in increased interest in the relationship between retinal vascular disease and choroid vascular structure [5, 6].

Several studies have found an increase in the subfoveal choroidal thickness (SFCT) of patients with BRVO that tends to decrease after anti-VEGF, or dexamethasone treatment [7–12]. This implies that the effects of retinal VEGF are not just confined to the retina, but also reach the choroidal structure beyond the barrier of retinal pigment epithelium (RPE) tight-junction. Although we could assume that choroidal vascularity reflects the severity of retinal diseases, there has been much debate regarding the suitability of using SFCT as a parameter to predict prognosis as SFCT varies due to age, gender, refractive errors and other factors [13]. However, a novel, more accurate choroidal structural analysis, called the choroid vascularity index (CVI) has now been introduced. This enables the ratio of the luminal area (LA) to the stromal area (SA) to be calculated by image binarization [13–15].

In the current study, we identified how the choroidal vasculature changed in eyes with BRVO and ME, by comparing diseased eyes with unaffected fellow and anti-VEGF treated eyes using the CVI. Furthermore, we performed several analyses to ascertain whether there were any correlations between the later VAs and choroidal measures (including unaffected fellow eyes) to assess the effectiveness of using CVI as a prognostic biomarker.

## Methods

### Study population

This retrospective observational study was conducted in the Department of Ophthalmology and Visual Science in Seoul St Mary’s Hospital and adhered to the tenets of the Declaration of Helsinki. All protocols were approved by the Institutional Review Board of Seoul St. Mary’s Hospital, The Catholic University of Korea Catholic Medical Center, South Korea. Owing to the retrospective nature and anonymized data of this study, the written informed consent procedures had been exempted under the provisions of the Institutional Review Board of Seoul St. Mary’s Hospital (KC20RISI0986).

Diseased eyes of patients diagnosed with monocular BRVO and ME at our clinic and their corresponding fellow eyes as controls were used. All participants were recruited between June 2017 and February 2020 at Seoul St. Mary’s Hospital in Korea, and a retrospective review of their medical records was performed. The exclusion criteria were as follows: (1) refractive errors of more than ± six diopters (as spherical equivalent), (2) eyes with a history of any ocular trauma, laser treatment, or intraocular surgery, (3) eyes with a history of intravitreal injections, (4) other systemic diseases that could affect the eye, including diabetes mellitus, (5) presence of other retinal diseases, including glaucoma, age-related macular degeneration, diabetic retinopathy, pachychoroid disease, or neurodegenerative disease (6) media opacity that could affect image quality, (7) any history of uveitis.

### Study protocol

Demographic data, medical history, and ophthalmologic history were recorded at the initial visit. All subjects underwent an ocular examination, which included a best-corrected visual acuity (BCVA) evaluation, non-contact pneumatic tonometry, slit-lamp microscopy, dilated fundus examination, and OCT. The initial and two years post disease onset VA using the Snellen chart were converted to the logarithm of the minimum angle of resolution scale (logMAR). Imaging was obtained with an SS-OCT device (DRI Triton, Topcon, Tokyo, Japan) using a 1050-nm wavelength light source, and a scanning speed of 100,000 A-scans/second. A 6-line radial pattern scan (1024 A-scans) centered on the fovea was performed for each eye.

### Image analysis

BRVO and ME was diagnosed when its typical characteristics were present in a fundus examination and the central macular thickness (CMT) was >300 μm. CMT was determined using a thickness map in the SS-OCT software. BRVO eyes with superficial hemorrhage involving central macular lesion which could considerably affect the CVI calculation were excluded from image analysis. To minimize the effect of disease periods on the CVI, we analyzed the OCT images of both eyes at the initial visit and the diseased eyes around two months after the first anti-VEGF treatment. All of the OCT images were evaluated by two experienced independent retinal specialists (Y-H.P. and M.K.) who were blinded to the other imaging findings and the patients’ clinical histories.

### Choroidal thickness measurement

Choroidal thickness was calculated using an automatic built-in software within the SS-OCT device. SFCT was determined by calculating the distance from the outer border of the RPE to the inner edge of the suprachoroidal space [16]. We measured SFCT manually at the foveal center using digital calipers provided by the SS-OCT software. Two experienced independent observers measured the SFCT, and the average value was utilized in the analysis to avoid inter-observer variation.

### Choroidal vascularity index assessment

**Fig 1:**
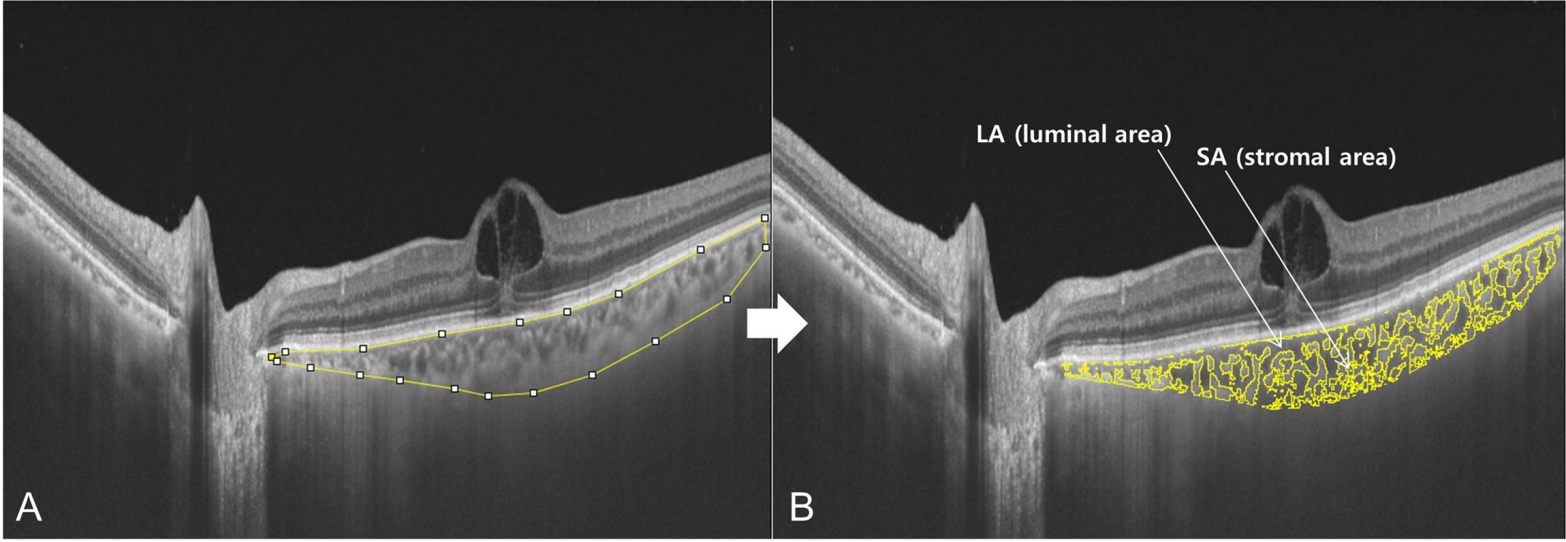
The choroidal vascularity index (CVI) measuring process with swept-source optical coherence tomography. The total choroidal area (TCA) was determined using the polygon selection tool in ImageJ software (A). Following image binarization using the Niblack auto local threshold tool, the stromal area (SA) within the selected TCA was highlighted. A distinction was set between the SA (the yellow part) and the luminal area (LA; the black part) (B). The CVI was calculated as the ratio of LA to TCA.

A 12 mm raster scan passing through the fovea was chosen for image binarization to obtain the CVI. It was segmented using the protocol described by Agrawal et al. [13], and image binarization was performed using Image J software (version 1.51; https://imagej.nih.gov/ij/). With the polygon selection tool, the total choroidal area (TCA) was selected, and regions of interest (ROIs) were added to the ROI manager (Fig 1A). After converting the image into 8 bit, a Niblack auto local threshold tool [17] was applied, which gave the mean pixel value with the standard deviation (SD) for all the points. After using the color threshold tool, the SA was highlighted and subsequently added to the ROI manager. Both of the initially selected polygonal TCA and highlighted SA were selected and merged through an "AND" operation in the ROI manager. This composition of areas was added to the ROIs manager as a third area. The LA in the polygon was determined by subtracting the third composite area (SA) from the total polygon area (TCA; Fig 1B). The ratio of LA to TCA was defined as the CVI.

### Statistical analysis

The statistical analysis was performed using the Statistical Package for the Social Sciences for Windows version 22.0 (SPSS, Inc, Chicago, IL). An exploratory analysis was conducted for all variables. The mean differences between the diseased, fellow, and anti-VEGF treated eyes were assessed using the Wilcoxon matched-pairs signed-rank test. A linear regression analysis was conducted between the choroidal parameters and logMAR VA measured two years post the onset of the disease. An univariate linear regression analysis was performed to analyze the effects of multiple factors associated with the CVI. Two-sided p-values of <0.05 were considered to be statistically significant.

## Results

**Fig 2:**
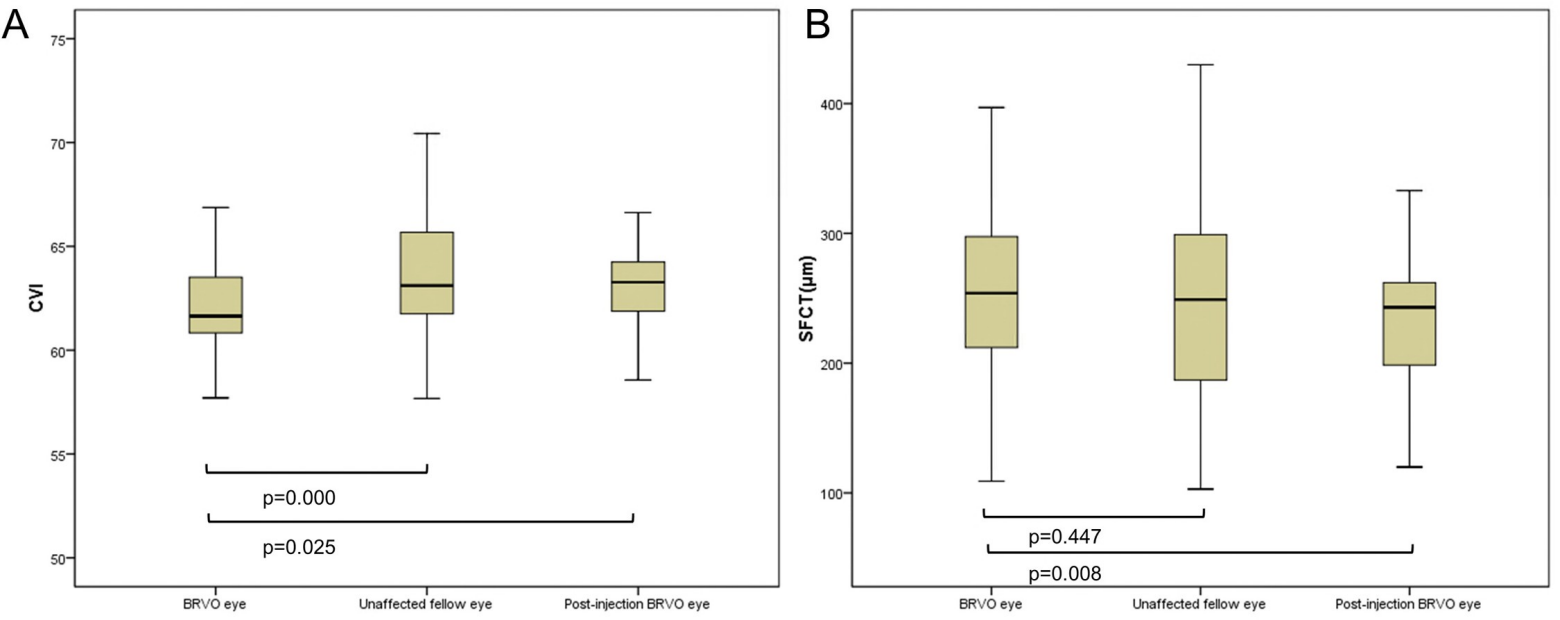
The choroidal vascularity index (CVI) and subfoveal choroidal thickness (SFCT) were measured to compare the branch retinal vein occlusion (BRVO), unaffected fellow, and post anti- vascular endothelial growth factor (VEGF) injected BRVO eyes. (A) The CVI of the BRVO eyes was significantly decreased compared to the unaffected fellow and post-injected eyes (all p<0.05). (B) The SFCT of the post anti-VEGF injected eyes was significantly decreased compared to the BRVO eyes (p<0.05), with no significant change between the BRVO and unaffected fellow eyes found (p = 0.447; Wilcoxon matched-pairs signed-rank test).

This study included 35 diseased and 35 unaffected contralateral (fellow) eyes from 35 patients diagnosed with monocular BRVO and ME at our clinic. Their 35 unaffected eyes were used as controls. Demographics and characteristics, including the macular and choroidal measurements (CMT, CVI, SFCT), are presented in Table 1. After intraocular anti-VEGF injections, macular edemas in 74.3% of total eyes recovered to less than 300 μm, and the mean value of CMT decreased from 494 μm to 285 μm. The mean CVI of the BRVO eyes was 62.16 ± 2.08%, which was significantly lower than that of the fellow (p<0.001) and post-injected BRVO eyes (p = 0.025). The mean SFCT of the BRVO eyes was 253.62 ± 66.61 μm, which was not significantly different when compared to the fellow eyes (p = 0.447), but significantly higher than post-injected BRVO eyes (p = 0.008; Fig 2).

**Table 1:** Demographics, characteristics and choroidal measurements of the study subjects.

	Total n = 35
Age, years	65.85 (±11.53)
Sex, male:female	16:17
Hypertension, n (%)	10 (28.6%)
Disease eye, OD:OS	18:17
Initial BCVA, decimal	0.40 (±0.24)
Initial IOP, mmHg	14.02 (±3.28)
Refraction, spherical equivalent	-0.82 (±2.73)
Initial CMT (μm)	494.34 (±134.67)
Post-injected CMT (μm)	285.74 (±73.62)
CVI (%)	62.16 (±2.08)
Fellow eye CVI (%)	63.71 (±3.34)
Post-injected CVI (%)	63.01 (±2.34)
SFCT (μm)	253.62 (±66.61)
Fellow eye SFCT (μm)	246.31 (±76.96)
Post-injected SFCT (μm)	234.28 (±68.80)

Data are presented as the mean (± SD) or a number (%), as appropriate.

BCVA, Best-corrected visual acuity; IOP, intraocular pressure; CMT, central macular thickness; CVI, choroidal vascularity index; SFCT, subfoveal choroidal thickness.

**Fig 3:**
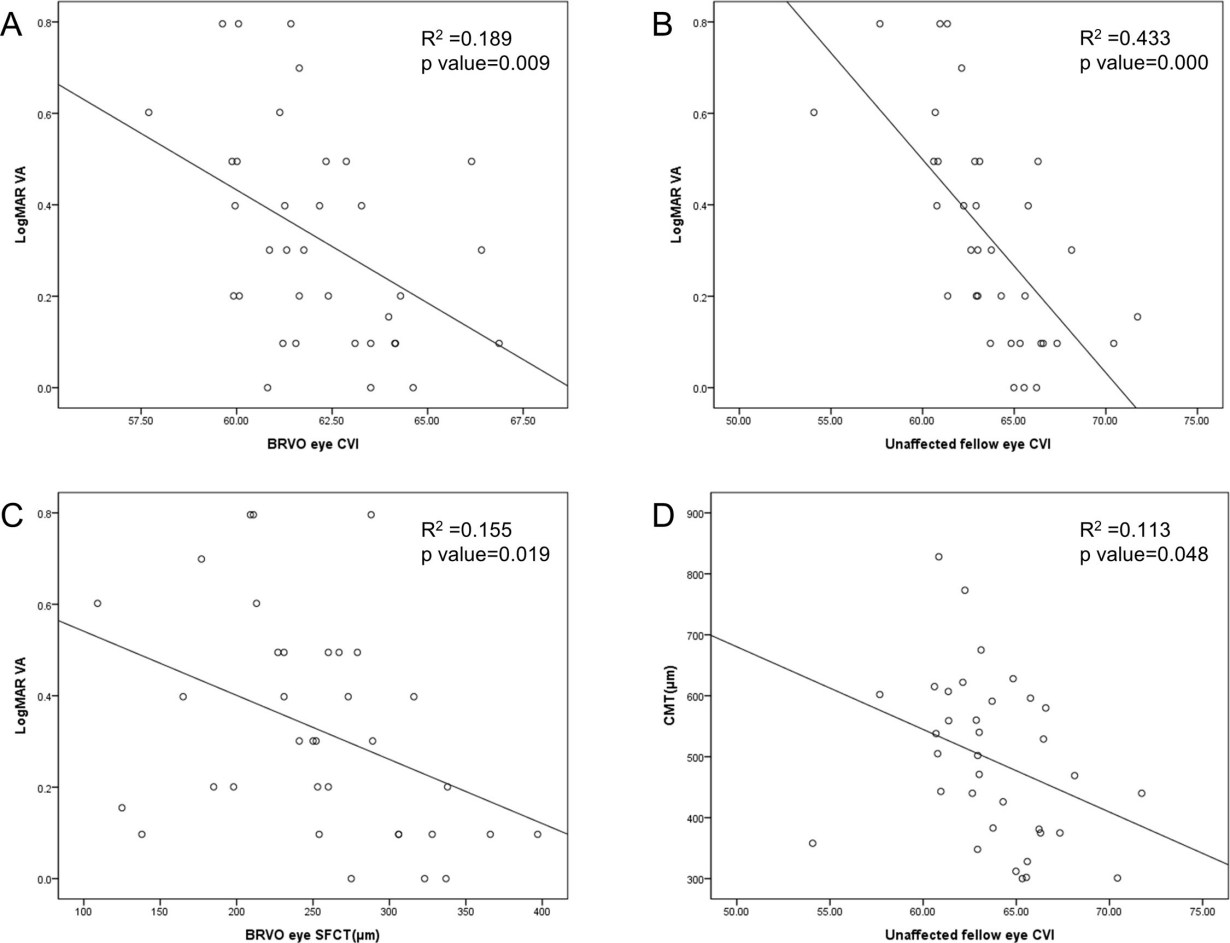
The linear regression analysis between the choroidal parameters and logarithm of the minimum angle of resolution scale (logMAR) visual acuity measured two years post the disease onset. The correlation between the choroidal vascularity index (CVI) of unaffected fellow eyes and the central macula thickness (CMT) of BRVO eyes. The R^2^ and p-values are presented. (B) The CVI of unaffected fellow eyes reveal the most prominent correlation value and a significant p-value; (A), (C) With the significant p-values, remarkable correlations are presented in the CVI and subfoveal choroidal thickness (SFCT) of the eyes with BRVO. (D) The CVI of unaffected fellow eyes shows a significant association with the CMT of diseased eyes.

The results of the linear regression analysis between the possible prognostic choroidal values and logMAR VA two years post disease onset were shown in Fig 3. The correlation between the CVI of unaffected fellow eyes and the CMT of BRVO eyes was also presented in Fig 3. The CVI of unaffected fellow eyes revealed the most prominent correlation (R^2^ = 0.433) and significant p-value (p<0.001; Fig 3B). Remarkable correlations were found in the CVI and the SFCT of BRVO eyes (R^2^ = 0.189, 0.155, respectively) with significant p-values (0.009, 0.019, respectively; Fig 3A and 3C). The CMT of diseased eyes were also significantly associated with the CVI of unaffected fellow eyes (R^2^ = 0.113, p = 0.048; Fig 3D).

A univariate analysis was performed to evaluate the factors that can affect the CVI values. The univariate regression analysis revealed that age, sex, BCVA, hypertension, intraocular pressure (IOP), and SFCT were not significantly associated with the CVI (p>0.05), except for CMT (p<0.05) (Table 2).

**Table 2:** Univariate regression analysis results.

	Univariate	
Variables	Standardize ß	p-value
Age, years	0.048	0.139
Sex, female	0.192	0.799
BCVA, logMAR	-0.837	0.452
Systemic hypertension	0.077	0.923
IOP, mmHg	-0.171	0.119
CMT, μm	-0.006	0.033
SFCT, μm	0.005	0.332

Linear regression analysis of the factors associated with the CVI. ß, regression coefficient. p-values that were statistically significant are highlighted in bold. BCVA, Best-corrected visual acuity; logMAR, logarithm of the minimum angle of resolution; IOP, intraocular pressure; CMT, central macular thickness; SFCT, subfoveal choroidal thickness.

## Discussion

In the present study, the univariate regression analysis showed no remarkable correlation between CMT and CVI (ß = -0.006), supporting the SS-OCT device helped to clearly visualize the choroid by having a longer center wavelength, minimizing the effect of edema-induced OCT signal attenuation, which could be obviously identified in Fig 1. A possible mechanism of the decreased CVI in the eyes with BRVO and ME eyes could be extracellular fluid shift from retina to choroid. Aribas et al. [18] considered a pressure effect of choroidal congestion as the main cause of decreased CVI in BRVO. They demonstrated that the rush of extracellular fluid toward choroids through outer retinal barrier was caused by the blockage of retinal venous outflow and facilitated by ischemia-induced Aquaporin 9. However, in a long-term perspective, further studies should be done to elucidate a more robust theory for the alterations of CVI in eyes with BRVO. The result of CVI reduction in BRVO patients with mean disease duration of more than 3 years in the Aribas et al. study might be related to choriocapillary (CC) changes because they also presented that of CC density reduction in optical coherence tomography angiography (OCTA).

The increased CVI after anti-VEGF treatment in our study could be assumed that diminished flow ingress from retina by the anti-VEGFs effects on the recovery of inner BRB functions might increase the CVI, even in the process of decreased choroidal blood flow and choroidal thickness by the anti-VEGF effects [19, 20]. In addition, the decreased perfusion pressure in choroids might serve as a compensator maintaining selectively large choroidal vessel volume by the autonomic nerve system, contributing to the improved CVI after anti-VEGF treatments [21]. The measurement of luminal area in the CVI calculation would be affected mostly by larger vessels, which have abundant smooth muscles and elastic fibers innervated by the autonomic nerve system. The SFCT values in our study were only slightly consistent with that of previous studies in that no significant change was found between the BRVO and fellow eyes, but SFCT decreased after anti-VEGF treatment. Similar to the present study, Okamoto et al. [22] also found that in patients with BRVO and ME, choroidal thickness and choroidal blood flow decreased significantly after the anti-VEGFs injection. The result of contrasting increase and decrease of CVI and CT after the treatment appeared to protect the vascular luminal area of choroid. In addition to the hypothesis of decreased retinal backflow and autonomic compensatory action, mentioned above, further studies might be needed to elucidate the effect of CC changes on the CVI in RVO due to the fact that more than half of the choroidal vessels in the EDI OCT image were choroidal veins which arose from CC [23]. The pattern that the post injection CVI did not increase to the level of the unaffected fellow eye might also imply the possibility of CC changes after anti-VEGF injection despite no significant difference between the post injected CVI and the fellow eyes’ CVI (p = 0.136).

For clinical application, we performed a linear regression analysis between the choroidal parameters and logMAR VA two years post disease onset. As the CVI of the contralateral fellow eyes showed the most prominent values among the coefficients of determination, the inherent CVI could be considered as a protective index in response to retinal ischemia. There could be some doubts about the correlation between CVI and choroidal microvasculature in that choroidal parameters like CVI or CT were not significantly correlated with the CC flow, which is regarded as the main blood supplier to RPE, in the previous study of health population [24]. However, Borrelli et al. proposed that the medium and large sized vessels in choroids could determine CC status with quadratic non-linear relationship [25]. According to the relationship showing U-shaped parabolic graph, it seems to be convincing that patients with a larger LA tend to have a larger CC flow especially in the condition of decreased LA and increased SA, possibly induced by the fluid shift phenomenon in BRVO and ME. Usually, the blood supply of the choroid is much higher than the amount of oxygen required for outer retina and RPE. However, In the case of diseases with a significant decrease in choroidal perfusion such as BRVO, it is suggested that CVI, which represents the larger blood vessels, may be an important indicator for blood supply to RPE or outer retina. In this respect, patients with a higher vessel proportion in the choroid were more likely to have an abundant supply for oxygen and nutrients, which would help to keep healthy RPE cells in the process of retinal fluid and VEGF transportation. Excess water and VEGFs in retina are normally transported to choroids via RPE cells with the paracellular fluid movement and the macromolecule transcytosis respectively, which could be formed by the ATP-dependent active process, transepithelial electrical potential and membrane trafficking [26]. This mechanism could be applied to the correlation between the diseased eye CMT and the fellow eye CVI. If individuals originally had a large amount of vascular component in their choroids, the removal of fluid and VEGF from retina might operate well, possibly reducing the severity of ME and maintaining the outer retinal integrity. Regarding the effect of VEGFs or anti-VEGFs on RPE permeability, there has been some controversy because the experimental conditions varied for each study [26–28]. Therefore, further researches for RPE permeability are also needed to settle a robust theory for the transportation of crucial elements via RPE in retinal ischemic condition. The correlation between the BRVO eye CVI and the VA 2 years post disease onset could be significant in the present study since the degree of fluid shift would be dependent on that of ischemia. The result of the BRVO eye SFCT and the VA 2 years post disease onset could also be understood in the same context as high values of CVI and those of CT has been closely associated [13].

There were some limitations to this study. A small number of patients were included in the analysis, and only a single macular scan was used to calculate the CVI. A 3D volume image could be measured using OCTA, which would enable the choroidal vessel components to be calculated as a volume rather than an area [29]. If combined with the new modalities that have enabled ophthalmologists to minimize signal attenuation induced by macular edema, OCT or OCTA could help to provide more accurate and meaningful information. In addition, as the CVI was manually calculated in our study, newly invented automatic calculation software may help to improve the reproducibility of the CVI measurements. To cover a wider range as possible, we selected the whole choroidal area on a macular scan. However, it might be more useful only to measure the subfoveal lesions. Also, due to the exclusion for BRVO ME with superficial hemorrhage involving macula, this study could not cover certain cases which might represent a significant clue for choroidal changes in the disease.

This study revealed the association between BRVO and CVI with SS-OCT for the first time. As it has prognostic value, the concept of using the CVI of the unaffected fellow eye could help to promote the discovery of more elaborate indexes or biomarkers for clinical application, along with the development of new modalities. Regarding the choroid vascular component as one of the crucial elements in determining the final visual outcome in retinal vascular disease, we have shown that choroidal measurements could considerably support retinal biomarkers in BRVO and ME.

In conclusion, the alteration of CVI in BRVO and ME suggests that choroidal vasculature might be affected by extracellular fluid shift and VEGF changes. The CVI of unaffected fellow eyes could be a useful supplementary prognostic biomarker in patients with monocular BRVO and ME.

## Supporting information

S1 File. Dataset (XLSX)
